# Investigation of a Tumor Location-Specific Therapeutic Strategy for Intrahepatic Cholangiocarcinoma

**DOI:** 10.31557/APJCP.2021.22.5.1485

**Published:** 2021-05

**Authors:** Hisashi Kosaka, Masaki Kaibori, Kosuke Matsui, Morihiko Ishizaki, Hideyuki Matsushima, Mitsugu Sekimoto

**Affiliations:** *Division of Hepatic Surgery, Department of Surgery, Kansai Medical University, Hirakata City, Japan. *

**Keywords:** Cholangiocarcinoma, lymph node excision, hepatectomy, biliary tract, margins of excision

## Abstract

**Objective:**

An optimal therapeutic strategy for intrahepatic cholangiocarcinoma (ICC) has not yet been determined. Herein we focused on intrahepatic tumor location and retrospectively analyzed tumor characteristics depending on location to elucidate a location-specific therapeutic strategy for ICC.

**Methods:**

Sixty-five ICC patients were divided into three groups based on the distance between the innermost portion of the tumor and portal vein branches observed on preoperative imaging: peripheral, intermediate and central ICC.

**Results:**

Median disease-specific survival (DSS) of the peripheral ICC was not reached, whereas median DSS was 32.9 months in intermediate ICC and 25.2 months in central ICC (p <0.05). Vascular invasion was observed in all groups (56-92%). Bile duct invasion to the first branch of the hepatic duct was more commonly observed in central ICC (43%) compared with the peripheral and intermediate ICC (0-8%). Lymph node metastasis was not observed in peripheral ICC, whereas it was frequently observed in intermediate and central ICC (39-44%). A Cox regression analysis revealed sufficient RDI (≥58.3%) of adjuvant chemotherapy (AC) significantly increased the length of DSS (HR: 0.205). Based on these data, we have proposed a location-specific therapeutic strategy as follows: peripheral ICC requires anatomical resection without lymphadenectomy; intermediate ICC requires anatomical resection with lymphadenectomy and sufficient doses of AC; and central ICC requires anatomical resection with extrahepatic bile duct resection, caudate lobectomy, lymphadenectomy, and sufficient doses of AC.

**Conclusion:**

We propose an intrahepatic tumor location-specific therapeutic strategy for ICC. This information could contribute to the appropriate therapeutic management of patients with ICC.

## Introduction

Intrahepatic cholangiocarcinoma (ICC) is the second most common primary liver cancer arising in the liver, making up about 10% of all cholangiocarcinoma cases (Buettner et al., 2017; Shaib et al., 2004). The incidence of ICC has increased over the past 3 decades, while that of extrahepatic cholangiocarcinoma has remained stable (Welzel et al., 2006). ICC is an aggressive cancer, and surgical treatment is considered to be the only potentially curative treatment; however, long-term outcomes are generally dismal, especially in patients with lymph node (LN) metastasis or positive surgical margins (Buettner et al., 2017; Ercolani et al., 2010; Lafaroet al., 2015; Endo et al., 2008; Edeline et al., 2019). Although 30-47% of ICC patients who undergo lymphadenectomy have LN metastasis, it is challenging to diagnose LN metastasis preoperatively, and the necessity of lymphadenectomy is not yet well defined (Zhang et al., 2020; Bektas et al., 2015; Mavros et al., 2014; Li et al., 2013). Rates of curative resection for patients with ICC have been reported to be 65-87% (Marubashi et al., 2014; Bektas et al., 2015; Reames et al., 2017). Appropriate preoperative surgical planning is indispensable for achieving curative resection; however, longitudinal intraductal tumor extension is also difficult to diagnose preoperatively (Yao et al., 2018; Ji et al., 2019).

Some investigators have noted that intrahepatic tumor location can predict prognosis (Marubashi et al., 2014; Yamashita et al., 2016; Orimo et al., 2018; Zhang et al., 2018). Tumor location may affect prognosis due to the different tumor origins between perihilar-sided ICC and peripheral-sided ICC (Orimo et al., 2018; Aishima and Oda, 2015). This histological heterogeneity may cause confusion regarding the optimal therapeutic strategy for ICCs. In this study, we focused on intrahepatic tumor location and retrospectively analyzed tumor characteristics and clinical outcomes depending on tumor location to evaluate a tumor location-specific therapeutic strategy for ICC using preoperative imaging findings.

## Materials and Methods

*Patients*

Clinical data of 65 consecutive patients with tumors that were histologically confirmed as ICC following hepatectomy between January 2008 and April 2020 at Kansai Medical University were retrospectively analyzed. Patients with perihilar cholangiocarcinoma with invasion to the liver parenchyma were excluded from this study. Patients who were preoperatively diagnosed with ICC underwent hepatectomy with regional LN dissection, whereas patients who were diagnosed with hepatocellular carcinoma (HCC) or metastatic liver tumors underwent hepatectomy without regional LN dissection. Pathologic staging was evaluated according to the sixth edition of the Liver Cancer Study Group of Japan (LCSGJ) and the eighth edition of the American Joint Committee on Cancer (AJCC) guidelines (Liao and Zhang, 2020). Postoperative complications were evaluated by Clavien-Dindo score (Dindo et al., 2004).

*Intrahepatic tumor location-specific preoperative categorization of ICC*

**Figure 1:**
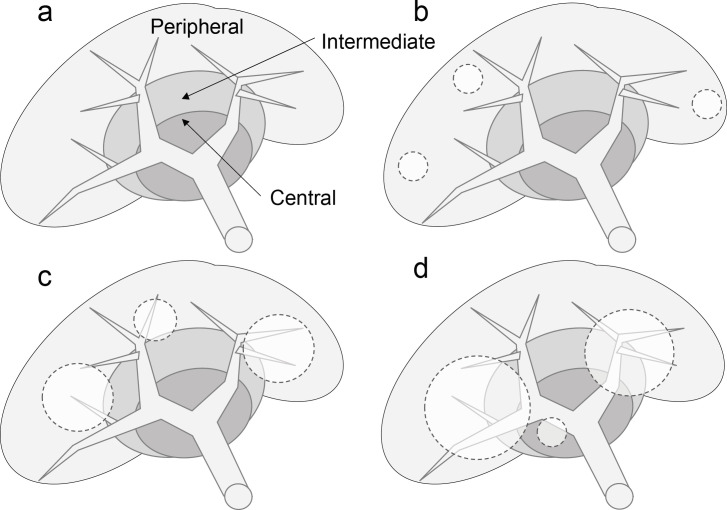
Proposed Criteria for Tumor Location-Dependent Stratification of Intrahepatic Cholangiocarcinoma. The liver was divided into three stratified zones based on the distance from the first or second portal vein branches (a). Tumor was displayed as dot circle. The location of the innermost portion of the tumor with respect to the hilus determined whether a tumor was a peripheral ICC (b), intermediate ICC (c), or central ICC (d)

Computed tomography (CT) and/or magnetic resonance imaging (MRI) was preoperatively performed to identify tumor location. The liver was divided into three areas based on the distance from the portal vein (PV) branches, as shown in Figure 1a. The area within 10 mm of the first portion of the PV (right and left PV) was defined as the central area. The area within 10 mm from the second portion of the PV (ie, the umbilical portion, anterior PV, and posterior PV) except the central area was defined as the intermediate area. The area except the intermediate and central areas was defined as the peripheral area. Tumors were stratified as peripheral ICC, intermediate ICC, or central ICC depending on the location of the innermost portion of the tumor relative to the hilus hepatis (Figure 1b-d). At the time of intrahepatic bile duct (IHBD) dilatation with no tumor mass formation observed, such as periductal infiltrating type tumors, MRCP and/or ERCP were performed to identify the innermost portion of the tumor relative to the hilus hepatis.

*Relative dose intensity (RDI) calculation*

The RDI of postoperative adjuvant chemotherapy (AC) for 6 months was calculated. A full dosage and cycles of AC for 6 months according to the manufacturer’s drug information was calculated as 100%. If patient had 1000 mg/m^2^ of gemcitabine (G) and 25 mg/m^2 ^of cisplatin (C) on days 1 and 8, every 3 weeks for eight cycles, RDI of GC was calculated as 100%. On the other hand, if patient had 20% of dose reduced GC biweekly (800 mg/m^2^ of G and 20 mg/m2 of C on days 1, every 2 weeks for 12 cycles), RDI was calculated as 60%. GC was administered postoperatively to ICC patients in our hospital, whereas tegafur/gimeracil/oteracil (S) or GS were given depending on the patient’s condition, considering renal function, allergic history, and adverse events (AEs) in response to GC. Data from 27 patients who completed 6 months of AC administration were evaluated, whereas the data of 4 patients who did not complete 6 months of AC by the early recurrence within 6 months after surgery were excluded. 

*Statistics*

Data are expressed as numbers with percentages or medians with interquartile ranges (IQRs). Continuous variables were compared with Mann-Whitney U test or Kruskal-Wallis test. Nominal scale data were examined using the chi-squared test. Kaplan-Meier curves were used to estimate median survival. Log-rank testing was performed to assess differences in disease-specific survival (DSS) and disease-free survival (DFS). Cox proportional hazards regression analysis was performed using a forward stepwise method to detect independent risk factors of DSS. Hazard ratios with 95% confidence intervals (CIs) were estimated. Receiver operating characteristic (ROC) curve analysis was performed to identify RDI cut-off values. Comparisons were considered statistically significant at p values <0.05. All statistical analyses were performed using the IBM SPSS 22 software package for Windows (IBM Japan Ltd., Tokyo, Japan).

*Ethical code*

This study was approved by the institutional review board of Kansai Medical University (Approval number: 2019322). This current study was performed in accordance with the Declaration of Helsinki.

## Results

*Background characteristics of ICC depending on tumor location*

Sixty-five ICC patients were stratified into peripheral ICC (25%), intermediate ICC (40%), and central ICC (35%) groups according to the location of their innermost tumors, which were preoperatively detected by CT, MRI, or ERCP/MRCP. The criteria for tumor location-specific ICC stratification are described in Figure 1. Background characteristics are listed in Table 1. The majority of patients were older (72 years) and male (71%). Hepatitis B virus infection was observed in 35% of patients, whereas hepatitis C virus infection was only present in 8%. Almost all patients were asymptomatic in the peripheral ICC (88%) and intermediate ICC (89%) groups, whereas 56% of patients in the central ICC group were symptomatic (p<0.05). While preoperative diagnoses matched postoperative histological diagnoses as ICC in the intermediate ICC (81%) and central ICC (78%) groups, preoperative diagnosis tended to be challenging in the peripheral ICC (50%) group; however, the difference was not statistically significant (p ≥0.05). Total bilirubin levels, Child-Pugh score, and indocyanine green (ICG) percentages were not different between the three groups (p ≥0.05), whereas levels of biliary enzymes, such as alkaline phosphatase (ALP) and γ-glutamyl transpeptidase (γ-GTP), increased from peripheral ICC to central ICC in a stepwise fashion (p <0.05). Low levels of albumin and high C-reactive protein (CRP) values were observed in the central ICC group (p <0.05). CA19-9 levels tended to be high in the intermediate ICC and central ICC groups, although the difference was not statistically significant (p ≥0.05). 

*Comparisons of surgical and postsurgical outcomes depending on tumor location*

Table 2 demonstrated comparisons of surgical and postsurgical outcomes depending on tumor location. Surgical procedures differed depending on intrahepatic tumor location. Anatomical resection was performed in all intermediate and central ICC patients, whereas it was conducted in only 56% of peripheral ICC patients. The majority of peripheral ICC patients underwent partial resection or segmentectomy (44%) without caudate lobectomy (0%) or extrahepatic bile duct resection (0%), whereas the majority of intermediate ICC patients underwent bisectionectomy (69%) without caudate lobectomy (15%) or extrahepatic bile duct resection (7.7%). In contrast, the majority of patients with central ICC underwent bisectionectomy (74%) or trisectionectomy (26%) with en bloc caudate lobectomy (48%) and extrahepatic bile duct resection (39.1%). Regional lymphadenectomy was performed in patients with preoperatively diagnosed intermediate ICC (62%) or central ICC (78%), whereas it was not routinely performed in patients with peripheral ICC (25%) (p <0.05). Operative time and blood loss increased in a stepwise fashion from the peripheral ICC to the central ICC groups (p <0.05). The proportion of patients with a Clavien-Dindo score -IIIa did not differ between groups (p ≥0.05), whereas postoperative hospital stay was significantly prolonged in the central ICC group (30 days) compared with the peripheral ICC (13 days) and intermediate ICC (12.5 days) groups. Adjuvant chemotherapy was administrated to 59% of patients in the entire cohort, without significant differences between the 3 tumor location groups (p ≥0.05).

*Histopathological characteristics depending on tumor location*

Table 3 demonstrated histopathological characteristics depending on tumor location. The majority of tumors were mass-forming type (69%) and moderately differentiated (60%), without significant differences between tumor location groups (p ≥0.05). Tumor size increased in a stepwise fashion from the peripheral ICC (33.5 mm) group to the central ICC (80.0 mm) group (p <0.05). Considerable vascular invasion was observed in the peripheral ICC group (56%), whereas it was observed roughly in almost all patients in the intermediate ICC (92%) and central ICC (87%) groups (p <0.05). Bile duct invasion into the first branch of the hepatic duct was rarely observed in the peripheral ICC (0%) and intermediate ICC (8%) groups, whereas it was observed in 43% of patients in the central ICC group (p <0.05). Regional LN metastasis was not observed in the peripheral ICC group (0%), whereas it was observed frequently among patients in the intermediate ICC (44%) and central ICC (39%) groups (p <0.05). Eighth edition AJCC Stage ≥IIIA disease was confirmed in a small percentage of patients in the peripheral ICC group (13%), whereas it was confirmed in greater percentages of patients in the intermediate ICC (58%) and central ICC (65%) groups (p <0.05).

*Comparison of survival depending on tumor location*

**Figure 2:**
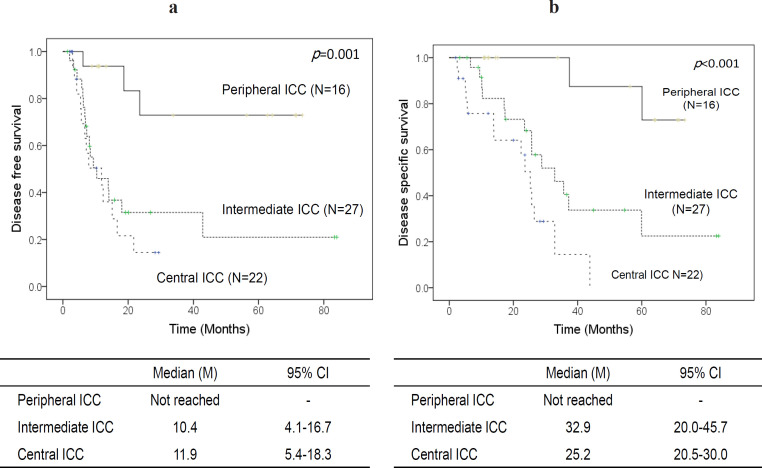
Comparison of Survival Depending on Tumor Location. Disease-free survival (a) and disease-specific survival (b) depending on tumor location are shown: peripheral ICC (black line, n=16) versus intermediate ICC (thick dots, n=27) versus central ICC (thin dots, n=22). ICC, Intrahepatic cholangiocarcinoma; CI, Confidence interval

DFS and DSS depending on tumor location are shown in Figure 2. Median DFS was not reached in the peripheral ICC group, whereas median DFS in the intermediate ICC and central ICC groups was poor at 10.4 and 11.9 months, respectively (p <0.05). Median DSS was also not reached in the peripheral ICC group, whereas median DSS decreased in a stepwise manner from 32.9 months in the intermediate ICC group to 25.2 months in the central ICC group (p <0.05). 

*Adjuvant chemotherapy-dependent survival in the intermediate ICC and central ICC groups*

**Figure 3:**
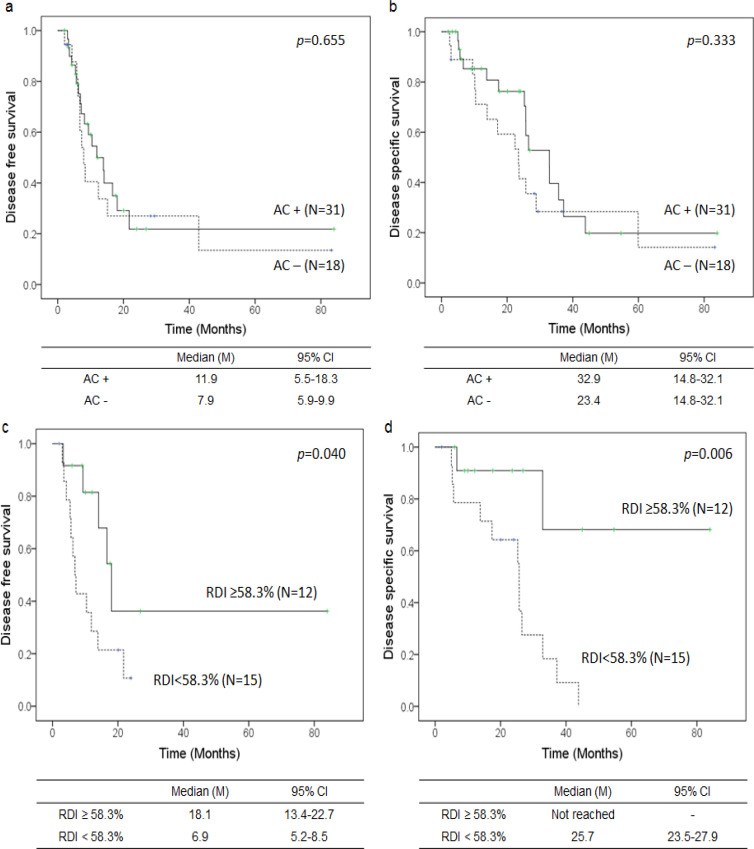
Correlations between Adjuvant Chemotherapy and Survival in a Mixed Cohort of Intermediate/Central ICC Patients. Disease-free survival (a) and disease-specific survival (b) depending on administration of AC in a mixed cohort of intermediate/central ICC (n=49) patients are shown: patients who received AC (black line, n=31) versus patients who did not receive AC (thick dots, n=18). Disease-free survival (c) and disease-specific survival (d) depending on the RDI in patients who completed 6 months of AC (n=27) are shown: patients with RDI ≥58.3% (black line, n=12) versus patients with RDI <58.3% (thick dots, n=15). AC, Adjuvant chemotherapy; RDI, Relative dose intensity; CI, Confidence interval

Overall, 65.8% of patients received GC as AC and 31.6% received S or GS. To evaluate the impact of AC on the prognosis of ICC patients, the associations between DFS and DSS and AC administration were evaluated in a mixed cohort of intermediate and central ICC patients (n=49). Log-rank testing revealed that AC did not influence DFS among patients with intermediate or central ICC (Figure 3a). Median DFS was 11.9 months in patients who received AC and 7.9 months in patients who did not receive AC (p >0.05). Similarly, AC did not affect DSS among patients with intermediate or central ICC (Figure 3b). Median DSS was 32.9 months in patients who received AC and 23.4 months in patients who did not receive AC (p >0.05). A supplementary analysis revealed that AC also did not influence DFS or DSS in patients with peripheral ICC (p >0.05 respectively, data not shown). ROC curve subgroup analysis of 27 patients who completed 6 months of AC was performed to determine the cutoff value of RDI for DSS (n=27). The RDI cutoff value was determined to be 58.3% (Area under the curve: 0.720, Confidence interval: 0.560 - 0.880, p =0.013), and patients were divided into RDI ≥58.3% (n=12) and RDI <58.3% (n=15) groups. Log-rank testing revealed that DFS in the RDI ≥58.3% subgroup was significantly prolonged compared with that in the RDI <58.3% subgroup (Figure 3c): median DFS was 18.1 months in patients with RDI ≥58.3% and 6.9 months in patients with RDI <58.3% (p <0.05). Additionally, DSS in the RDI ≥58.3% subgroup was significantly prolonged compared with that in the RDI<58.3% subgroup (Figure 3d). Median DSS was not reached in patients with RDI ≥58.3%, whereas median DSS was 25.7 months in patients with RDI <58.3% (p <0.05). Cox regression analysis was performed to evaluate factors associated with DSS (Table 4). Sufficient RDI (≥58.3%) significantly increased the length of DSS, with a hazard ratio of 0.205, whereas involvement of regional lymph nodes (N) according to the AJCC staging system increased the risk of DSS, with a hazard ratio of 16.5 (p <0.05). 

**Table 1:** Background Characteristics of ICC Depend on the Tumor Location

	Over all	Peripheral ICC	Intermediate ICC	Central ICC	p value
N	65	16 (25)	26 (40)	23 (35)	-
Age	72.0 (66.0-76.5)	72.0 (68.0-75.5)	71.5 (69.8-79.5)	71.0 (62.0-74.0)	0.326
Gender, Male	46 (71)	12 (75)	18 (69)	16 (70)	0.912
HBV	23 (35)	7 (44)	7 (27)	9 (39)	0.486
HCV	5 (8)	2 (13)	1 (4)	2 (9)	0.578
Asymptomatic	47 (72)	14 (88)	23 (89)	10 (44)	*0.001
Preoperative diagnosis as ICC	47 (72)	8 (50)	21 (81)	18 (78)	0.07
Bile drainage	16 (25)	1 (6)	7 (27)	8 (35)	0.119
PTPE	3 (5)	0 (0)	1 (4)	2 (9)	0.432
NAC	5 (8)	0 (0)	1 (4)	4 (17)	0.085
ALB (g/dL)	4.0 (3.8-4.3)	4.1 (3.8-4.5)	4.0 (3.8-4.3)	3.8 (3.0-4.1)	*0.033
Total bilirubin (mg/dL)	0.6 (0.5-0.8)	0.6 (0.6-0.7)	0.6 (0.5-0.8)	0.6 (0.5-0.9)	0.799
AST (U/L)	26.0 (20.0-32.0)	24.5 (18.5-29.8)	26.0 (21.5-31.8)	27.0 (20.0-32.0)	0.617
ALT (U/L)	20.0 (14.0-27.0)	16.5 (13.3-26.5)	21.0 (14.8-27.0)	22.0 (14.0-28.0)	0.679
ALP (U/L)	265.0 (216.5-387.5)	230.0 (193.0-286.0)	261.0 (213.5-382.8)	333.0 (239.0-544.0)	*0.010
γ-GTP (U/L)	64.0 (30.5-132.0)	28.0 (19.8-62.5)	59.0 (40.3-109.8)	138.0 (68.0-359.0)	*<0.001
CRP (mg/dL)	0.14 (0.09-0.63)	0.09 (0.03-0.22)	0.13 (0.09-0.40)	0.36 (0.11-1.74)	*0.019
Platelet (×104μL)	19.4 (14.3-24.9)	18.3 (13.5-22.7)	19.6 (15.6-23.6)	21.8 (14.8-26.1)	0.394
PT (%)	92.4 (83.7-104.9)	91.2 (84.1-107.0)	94.2 (82.8-104.7)	90.4 (83.3-101.7)	0.971
Child-Pugh A	62 (96)	15 (94)	26 (100)	21 (91)	0.329
ICG (%)	10.2 (7.7-14.8)	9.6 (6.5-12.9)	9.7 (8.0-12.1)	11.5 (7.6-16.2)	0.53
CEA (ng/mL)	3 (1.8-4.7)	3.2 (1.7-4.4)	3.1 (1.8-4.9)	2.8 (2.1-6.4)	0.722
CA19-9 (U/mL)	31.3 (14.0-124.5)	23.1 (4.0-46.5)	43.6 (13.7-211.0)	38.8 (22.5-214.1)	0.107

ICC, Intrahepatic cholangiocarcinoma; HBV, Hepatitis B virus; HCV, Hepatitis C virus; PTPE, Percutaneous transhepatic portal vein embolization; NAC, Neoadjuvant chemotherapy; ALB, Albumin; AST, Aspartate transaminase; ALT, Alanine transaminase; ALP, Alkaline phosphatase; γ-GTP, γ-glutamyl transpeptidase; CRP, C-reactive protein; PT, Prothrombin time; ICG, Indocyanine green; CEA, Carcinoembryonic antigen; CA19-9 , Carbohydrate antigen 19-9; *p<0.05

**Table 2:** Comparisons of Surgical and Postsurgical Outcomes Depending on Tumor Location

	Over all	Peripheral ICC	Intermediate ICC	Central ICC	p value
N	65	16 (25)	26 (40)	23 (35)	-
Anatomical resection	58 (89)	9 (56)	26 (100)	23 (100)	*<0.001
Resection range					*<0.001
Segmentectomy or partial hepatectomy	8 (12)	7 (44)	1 (4)	0 (0)	
Sectionectomy	11 (17)	6 (38)	5 (19)	0 (0)	
Bisectionectomy	38 (59)	3 (19)	18 (69)	17 (74)	
Trisectionectomy	8 (12)	0 (0)	2 (8)	6 (26)	
Caudate lobectomy	15 (23)	0 (0)	4 (15)	11 (48)	*0.001
Extrahepatic bile duct resection	11 (16.9)	0 (0)	2 (7.7)	9 (39.1)	*0.002
Portal vein or hepatic artery reconstruction	5 (8)	0 (0)	1 (4)	4 (17)	0.085
Laparoscopic surgery	11 (17)	6 (38)	5 (19)	0 (0)	*0.008
Regional lymphadenectomy	38 (59)	4 (25)	16 (62)	18 (78)	*0.004
Operative time (min)	387.0 (304-527)	271.0 (215-354)	382.5 (323-428)	549.0 (405-664)	*<0.001
Blood loss (ml)	933.0 (489-1209)	457.0 (62-932)	885.0 (501-1138)	1199.0 (892-1912)	*0.001
Blood transfusion	32 (49)	4 (25)	15 (58)	13 (57)	0.082
Clavien-Dindo score, a and more	22 (34)	4 (25)	7 (27)	11 (48)	0.21
Hospital stays (days)	19.0 (10.5-31.0)	13.0 (8.3-22.3)	12.5 (11.0-24.3)	30.0 (16.0-75.0)	*0.005
90 days mortality	2 (3)	0 (0)	0 (0)	2 (9)	0.179
Adjuvant chemotherapy	38 (59)	7 (44)	17 (65)	14 (61)	0.369

ICC, Intrahepatic cholangiocarcinoma; *p<0.05

**Table 3:** Histopathological Characteristics Depending on Tumor Location

	Over all	Peripheral ICC	Intermediate ICC	Central ICC	p value
N	65	16 (25)	26 (40)	23 (35)	-
Tumor type					0.072
Mass forming	45 (69)	14 (88)	20 (77)	11 (48)	
Periductal infiltrating	14 (22)	1 (6)	4 (15)	9 (39)	
Intraductal growth	6 (9)	1 (6)	2 (8)	3 (13)	
Differentiation					0.667
Well	17(26)	6 (38)	6 (23)	5 (22)	
Moderate	39 (60)	7 (44)	17 (66)	15 (65)	
Poor	9 (14)	3 (19)	3 (12)	3 (13)	
Tumor size	50.0 (32.0-80.0)	33.5 (21.0-45.0)	48.0 (32.8-72.5)	80.0 (45.0-100.0)	*0.002
Multiple tumor	8 (12)	0 (0)	2 (8)	6 (26)	*0.033
Serosal invasion	22 (34)	2 (13)	10 (39)	10 (44)	0.108
Vascular invasion	53 (82)	9 (56)	24 (92)	20 (87)	*0.010
Bile duct invasion					*<0.001
2^nd^ branch of hepatic duct	14 (22)	0 (0)	1 (4)	13 (57)	
1^st^ branch of hepatic duct	9 (14)	0 (0)	2 (8)	7 (30)	
Common bile duct	3 (5)	0 (0)	0 (0)	3 (13)	
Lymph node metastasis**	14/38 (37)	0/4 (0)	7/16 (44)	7/18 (39)	0.26
Resection margin status, R0	53 (82)	14 (88)	23 (89)	16 (70)	0.376
8^th^ AJCC Stage A and more	32 (49)	2 (13)	15 (58)	15 (65)	*0.003
6^th^ LCSGJ Stage and more	49 (75)	7 (44)	23 (89)	19 (83)	*0.003

ICC, Intrahepatic cholangiocarcinoma; AJCC, American joint committee on cancer; LCSGJ, Liver cancer study group of Japan; *p<0.05; ** Regional lymph node metastasis was calculated as lymph node positive patients number among lymph node dissected patients.

**Table 4:** COX Regression for Disease Specific Survival of Intermediate and Central ICC with Adjuvant Chemotherapy (N=27)

	Hazard Ratio	95% CI	p value
Age	-	-	0.257
Gender, male	-	-	0.432
Trisectionectomy	-	-	0.123
Extrahepatic bile duct resection			0.437
Clavien-Dindo score, a and more	-	-	0.169
AJCC, T stage			0.709
AJCC, N stage	16.483	1.937-140.241	*0.010
Resection margin, positive			0.829
Duration till adjuvant chemotherapy	-	-	0.899
RDI ≥ 58.3%	0.205	0.043-0.976	*0.047

ICC, Intrahepatic cholangiocarcinoma; CI, Confidence interval; RDI, Relative dose intensity; AJCC, American joint committee on cancer; LCSGJ, Liver cancer study group of Japan; *p<0.05

**Figure 4:**
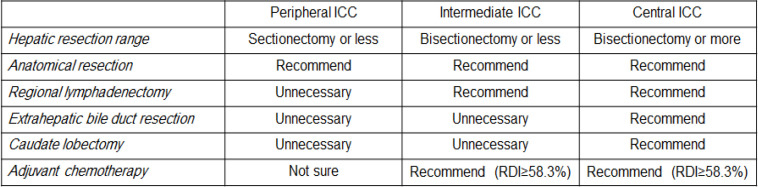
Tumor Location-Specific Therapeutic Strategy for Intrahepatic

## Discussion

ICC is an aggressive cancer, and surgical treatment is considered to be the only potentially curative treatment (Buettner et al., 2017; Ercolani et al., 2010; Endo et al., 2008; Orimo et al., 2018). ICC arising from various sites of the bile ducts requires individual surgical procedures depending on tumor location to achieve curative resection, such as extended hepatectomy, regional lymphadenectomy, en bloc extrahepatic bile duct resection, and caudate lobectomy (Buettner et al., 2017; Ercolani et al., 2010; Mavros et al. 2014; Yoh et al., 2019). However, it is hard to determine the longitudinal intraductal extension of tumors and LN metastasis preoperatively (Marubashi et al., 2014; Ji et al., 2019). An intrahepatic tumor location-specific therapeutic strategy has not yet been established (Li et al., 2013; Yoh et al., 2019). An individual therapeutic strategy for ICC should be preoperatively planned to achieve curative resection. We therefore analyzed location-specific histological characteristics to assess the utility of each procedure depending on intrahepatic tumor location.

Due to the connection between the caudate bile duct and the first portion of the hepatic duct, major hepatectomy with en bloc caudate lobectomy is standard treatment for perihilar cholangiocarcinoma (Endo et al., 2008; Sugiura et al., 2007; Tashiro et al., 1993). In this study, 39% of central ICC cases were periductal infiltrating type, and 43% of central ICC cases invaded the first branch of the hepatic duct, indicating that extrahepatic bile duct resection with en bloc caudate lobectomy was required for curative resection. In contrast, the majority of peripheral ICC and intermediate ICC cases were mass-forming type and rarely invaded the first branch of the hepatic duct (0-8%), indicating that neither en bloc extrahepatic bile duct resection nor en bloc caudate lobectomy was required. The difference in aggressiveness of bile duct invasion depending on intrahepatic tumor location has been previously explained: perihilar-sided ICCs originate from peribiliary gland cells caused periductal infiltrating type ICC, which demonstrate longitudinal tumor extension along the large bile duct, whereas peripheral-sided ICCs originate from the canals of Hering and interlobular bile ducts, causing mass-forming type ICC (Aishima and Oda, 2015).

Routine regional lymphadenectomy during ICC resection remains controversial (Li et al., 2013; Zhang et al., 2020; Yoh et al., 2019). Rates of regional lymphadenectomy among resected ICC cases have been variously reported as 43-98%, 21-47% of whom had LN metastasis (Mavros et al., 2014; Bektas et al., 2015; Li et al., 2013). Previous reports strongly recommended routine regional lymphadenectomy due to the considerable number of LN metastases observed during ICC resection (Lafaro et al., 2015). Other investigators have recommended routine regional lymphadenectomy for accurate staging (Zhang et al., 2020). A propensity score-matching study also noted that routine regional lymphadenectomy contributed to better prognoses in ICC patients who were not suspected preoperatively to have LN metastasis (Yoh et al., 2019). In this study, we proposed selective regional lymphadenectomy for intermediate ICC and central ICC cases. A total of 39-44% of LN metastases were observed in patients with intermediate and central ICC, whereas no metastases were observed in patients with peripheral ICC. Additionally, regional and distant LN recurrence was rarely observed in patient with peripheral ICC (6%, data not shown). Peripheral-sided ICC is considered to have similar characteristics as HCC and rarely invades LNs (Orimo et al., 2018; Aishima and Oda, 2015). Previous reports that referred to significantly low rates of LN metastasis in peripheral-sided, small ICCs support the present results (Marubashi et al., 2014; Orimo et al., 2018).

Recent report advocated anatomical resection was associated with better survival outcomes compared with non-anatomical resection in ICC patients with AJCC stage B or tumor without vascular invasion (Si et al. 2019). In this study, AJCC Stage ≤ disease was confirmed in 87% of patients in the peripheral ICC group, and percentage of patients without vascular invasion were 44%. Taken together, these results indicated considerable number of patients in the peripheral ICC group might require anatomical resection. Additionally, intermediate and perihilar ICC were closely located to 1^st^ or 2^nd^ branches of PV, therefore anatomical resection could be unavoidable.

Therapeutic efficacy of adjuvant chemotherapy following resection with curative intent in cholangiocarcinoma failed to be demonstrated in the PRODIGE 12-ACCORD 18 and BILCAP trials, which included 19-44% ICC patients among those with localized biliary tact cancers (Edeline et al., 2019; Primrose et al., 2019). The present results also demonstrated a lack of survival benefit among ICC patients treated with AC. However, in this study, we first noted the importance of RDI during AC for intermediate and central ICC, whereas peripheral ICC demonstrated a better prognosis regardless of AC and its RDI. It is significantly difficult to complete the scheduled full dose of AC after major hepatectomy regardless of regimen (Kainuma et al., 2015; Kobayashi et al., 2019). whereas intermediate ICC and central ICCs commonly require major hepatectomy. The present results indicate that a sufficient RDI (≥58.3%) was critical for achieving efficacy in this cohort of ICC patients with a poor prognosis.

In this study, we have divided the liver into 3 stratified zones—peripheral, intermediate, and central—based on portal vein branches. Histological characteristics and therapeutic outcomes were analyzed depending on innermost tumor locations. As a result of this investigation, we have determined intrahepatic tumor location-specific prognoses and have proposed an intrahepatic tumor location-specific therapeutic strategy for ICC as follows (Figure 4). Peripheral ICC requires sectionectomy or other anatomical resections; intermediate ICC requires bisectionectomy or lesser with regional lymphadenectomy and sufficient doses of AC; and central ICC requires bisectionectomy or greater with en bloc extrahepatic bile duct resection, en bloc caudate lobectomy, regional lymphadenectomy, and sufficient doses of AC. 

ICCs are histologically heterogeneous, depending on tumor location, between perihilar-sided ICCs and peripheral-sided ICC (Orimo et al., 2018; Aishima and Oda, 2015). Although intrahepatic tumor location-specific prognoses have been reported (Marubashi et al., 2014; Yamashita et al., 2016; Orimo et al., 2018), this is the first report of an intrahepatic tumor location-specific therapeutic strategy for ICC. A limitation of this work is that it is a retrospective study conducted in a single institution. We have therefore initiated a multicenter external validation study to confirm the utility of this tumor location-specific strategy. An intrahepatic tumor location-specific therapeutic strategy may contribute to optimal decision-making in ICC therapeutic management. 

## Author Contribution Statement

HK designed and performed this study. MK and MS edited the manuscript and supervised. KM, MI and HM worked on data acquisition and interpretation of data.
